# Early Life Disease Programming during the Preconception and Prenatal Period: Making the Link between Stressful Life Events and Type-1 Diabetes

**DOI:** 10.1371/journal.pone.0011523

**Published:** 2010-07-09

**Authors:** Jasveer Virk, Jiong Li, Mogens Vestergaard, Carsten Obel, Michael Lu, Jørn Olsen

**Affiliations:** 1 Department of Epidemiology, School of Public Health, University of California Los Angeles, Los Angeles, California, United States of America; 2 Department of Epidemiology, Institute of Public Health, Danish Epidemiology Science Centre, Aarhus University, Aarhus, Denmark; 3 Research Unit for General Practice, Aarhus University, Aarhus, Denmark; 4 Department of General Practice, Institute of Public Health, Aarhus University, Aarhus, Denmark; 5 Department of Obstetrics and Gynecology, University of California Los Angeles, Los Angeles, California, United States of America; Universidad Nacional Mayor de San Marcos, Peru

## Abstract

**Background:**

To assess the risk of developing Type-1 diabetes among children who were exposed to maternal bereavement during the prenatal or 1-year preconception period.

**Methods:**

We identified N = 1,548,746 singleton births born in Denmark between January 1^st^ 1979 through December 31^st^ 2004, and their next of kin. Altogether, 39,857 children were exposed to bereavement during their prenatal life. The main outcome of interest was hospitalization for type-1 diabetes (ICD 8: 249; ICD 10: E10).

**Results:**

We found the strongest association for type-1 diabetes among children exposed to traumatic father or sibling deaths (aIRR: 2.03, 1.22–3.38); the association was mainly seen for girls (aIRR: 2.91, 1.61–5.26).

**Conclusions:**

We found evidence to suggest that female fetuses exposed to severe prenatal stress are at increased risk for developing type-1 diabetes.

## Introduction

Diabetes is one of the most common chronic diseases among children; the increase in frequency of type-1 diabetes in youth has been among the most concerning aspects of the evolving epidemic. Incidence of type-1 diabetes varies with age, for boys it peaks at 12–13 years, and girls around 9–12 years [1]. A recent analysis conducted in 17 European countries suggests that cases of type-1 diabetes in children will double between 2005 and 2020, and that prevalent cases younger than 15 years will increase by 70%. Denmark has one of the highest reported incidences of type-1 diabetes in Europe (17.4 per 100,000), a rate predicted to increase annually by 3.2% [2].

Certain genetic factors have been associated with type-1 diabetes; but the observed changes in incidence over time cannot be attributable to susceptible genes alone, only in combination with environmental factors. Functions of isolated genes include insulin production and metabolism, protection from β-cell apoptosis, immunity and other unknown factors [3]. The role of inflammation in insulitis as a biological response to infection and β-cell loss has been studied in animal models. This process is not clearly understood but it must be induced early in life, where some combination of genetic and environmental factors operate to activate both endogenous and exogenous ligands of pattern-recognition receptors that induce islet inflammation and death of pancreatic β-cells. This is followed by a second process where insulitis is amplified through interplay between immune cells and β-cells, and third process where inflammatory mediators contribute to prolonged suppression and death of β-cells, or promotion, survival and proliferation of β cells [4].

The link between environmental and lifestyle factors such an increased weight and height [5], caesarean section deliveries [6] and early childhood infections [7], [8] and type-1 diabetes have all been assessed. Less is known about factors during pregnancy that may affect risk of type-1 diabetes, however high birth weight has been found to be associated with a slightly elevated risk for type-1 diabetes in a number of reports and also in a recent meta analysis [9]. The high incidence of type-1 diabetes in Denmark, and detailed national registers provides a unique opportunity for diabetes research. We hypothesized that exposure to stressful life events related to bereavement in the prenatal period, may increase the risk of type-1 diabetes. Bereavement was our indicator of stress since it almost uniformly induces severe stress and glucocorticoid production [10]–[12], and can be accurately ascertained using Danish Civil Registration system. The aim of this paper is to assess the risk of type-1 diabetes among children whose mothers were exposed to bereavement during the pre-natal or preconception period using a nationwide Danish cohort.

## Methods

**Figure 1:**
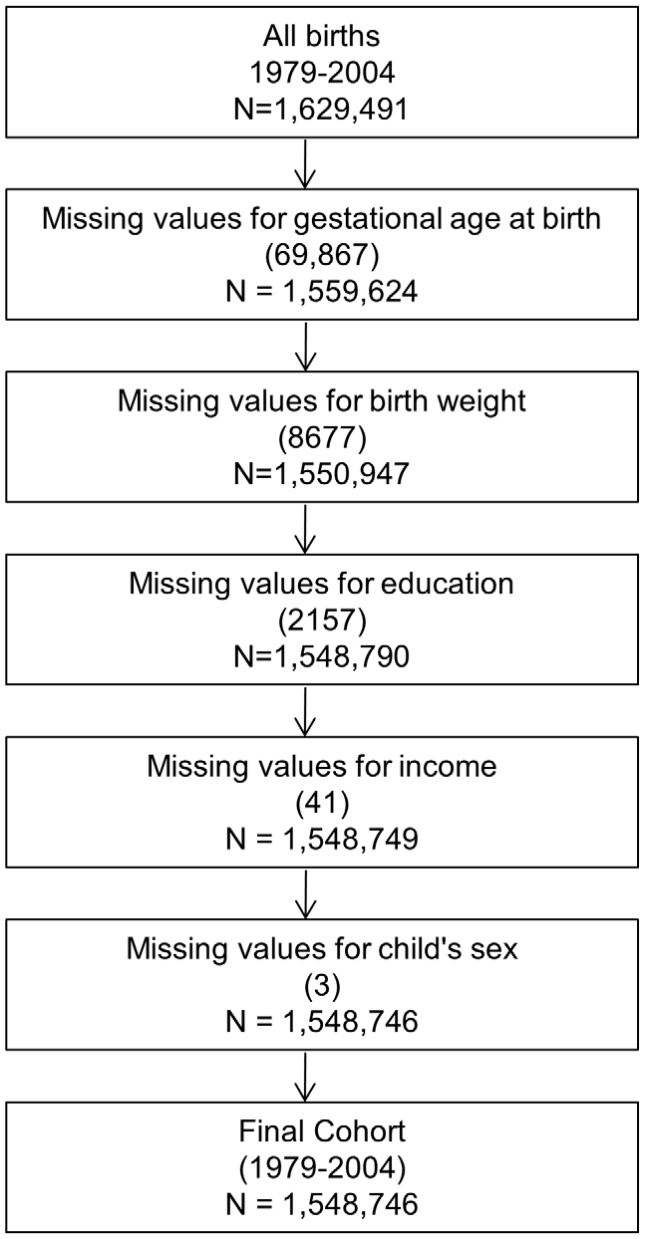
Flow chart for missing data.

Our results are based on a population based follow-up study on Danish national registers. We used data from the Danish Civil Registration System (CRS) to identify singleton births in Denmark born January 1^st^ 1979 through December 31^st^ 2004, N = 1,548,746 (see figure 1 for a flow chart of missing variables) and their next of kin (mother, father, siblings, mother's parents, and mother's siblings). All live born children and new residents in Denmark are assigned a unique civil personal registration (CPR) number, allowing accurate linkage of individual level data between registries.

Information on birth outcomes, such as gestational age at birth and birth weight, were obtained from the Danish Medical Birth Registry which has been computerized since 1973. Annual information on maternal education, residence, and income was retrieved from the Fertility Database at Statistics Denmark, available since 1979. The date of conception was estimated by the date of birth minus gestational age, and the prenatal exposure period included from 12 months before conception until the birth of the child. We categorized children as exposed to bereavement during prenatal life if their mothers lost a child, husband, sibling or parent during the prenatal period, ascertained via the death registry; all the remaining children were included in the unexposed cohort. Cohort members were followed from birth until first diagnosis of diabetes, death, emigration, or December 31^st^ 2004, whichever came first.

Diabetic status of children and their parents was ascertained from the National Hospital Registers, which holds information on all discharges from Danish hospitals since 1977; outpatients have been included in the register since 1995. Diagnostic information was based on the Danish version of the International Classification of Disease (ICD) 8^th^ revision, from 1979 to 1993 (249) and 10^th^ revision, from 1994 onwards (E10). We also looked for additional cases from the Pharmaco-epidemiological Prescription Database, in operation since 1995, using the ATC codes for insulin analog drugs (A10A), though there was almost 100% overlap with these cases and those found in the hospital register. If an individual has a diagnosis for both type-1 and type-2 diabetes, they were classified as type-1 since the later is more often misclassified than the former. Also, type-1 diabetes is a more serious disease than type-2 and therefore probably better diagnosed.

We estimated incidence rate ratios (IRRs) from birth using log-linear poisson regression models and used person-years as the offset variable. The analysis was performed using PROC GENMOD in SAS version 9.1.3. Unadjusted and adjusted models were generated. Adjusted estimates of IRRs (aIRR) included maternal age (≤18, 19–34, 35–40, 41+); residence (Copenhagen, cities with over 100,000 inhabitants, and other); income (1^st^, 2^nd^, 3^rd^, and 4^th^ quartile); maternal education (primary, secondary, high); parental diabetes status (any hospitalization diabetes ICD code); marital status (married, not married); gestational age at birth (<32 weeks, 32–36 weeks, 37+ weeks); birth weight (<1500 grams, 1500–2500 grams, 2500+ grams); sibling order (1,2,3,4+); calendar year (1979–1989, 1990–1999, 2000–2004) and offspring's sex (male, female). Age, residence, calendar period, maternal education, maternal income and parental marital status were treated as time-dependent variables as they were extracted from the birth year of the offspring.

To examine the association between timing of the bereavement and diabetes, we categorized exposed children by prenatal trimesters. To examine the relationship between type of bereavement and diabetes, we categorized exposed children by relationship to the deceased. We further categorized the cause of death by traumatic death (unexpected causes: ICD-8 codes 7950–7959, ICD-10 codes R95–R97; motor vehicle accidents: ICD-8 codes 8100–8230, ICD-10 codes V01–V89; suicide: ICD-8 codes 950–959, ICD-10 codes X60–X84; and other accidents and violence: ICD-8 codes 800–807, ICD-10 codes V90–V99, W00–X59, X85–Y89); and death from other causes. This study was approved by the Institutional Review Board at the University of California at Los Angeles. Individual written consent was not obtained from study participants as there was no record linking the subjects to identifying information.

## Results

We identified 39,587 children exposed to bereavement during or before their prenatal life. Children in our study were followed between 2 to 27 years. Exposed and unexposed children were comparable on most background characteristics although mother's of bereaved children were slightly older and had more children, something we expected to see among those who had lost a child (*see* *table 1*).

**Table 1:** Demographic variables for exposed and unexposed mothers (1979–2004).

		Exposed N = 39,857 (%)	Unexposed N = 1,508,889 (%)
Maternal age	≤18	315 (0.8)	17,857 (1.2)
	19–34	33,889 (85.0)	1,326,805 (87.9)
	35–40	5,264 (13.2)	152,277 (10.1)
	41+	389 (1.0)	11,950 (0.8)
Paternal age	≤18	694 (1.7)	26,359 (1.8)
	19–34	27,967 (70.2)	1,119,285 (74.2)
	35–45	10,422 (26.2)	337,333 (22.4)
	46+	774 (1.9)	25,912 (1.7)
Gestational age	<32	327 (0.6)	9,431 (0.6)
	32–36	2,154 (4.5)	68,137 (4.5)
	37+	37,376 (94.9)	1,431,321 (94.9)
Birth weight	<1500	8,795 (0.8)	321 (0.6)
	1500–2500	66,346 (5.3)	2,117 (4.4)
	>2500	1,433,748 (93.9)	37,419 (95.0)
Birth order	1	16,431 (41.2)	822,387 (54.5)
	2	15,461 (38.8)	503,300 (33.4)
	3	6,108 (15.3)	146,052 (9.7)
	4+	1,857 (4.7)	37,150 (2.4)
Income	1st quartile	8,914 (22.4)	376,265 (24.9)
	2nd quartile	16,592 (41.6)	584,169 (38.7)
	3rd quartile	11,346 (28.5)	435,578 (28.9)
	4th quartile	3,005 (7.5)	112,877 (7.5)
Education	Primary	16,791 (42.1)	637,143 (42.2)
	Secondary	12,703 (31.9)	479,195 (31.8)
	High	10,363 (26.0)	392,551 (26.0)
Marital status	Not married	16,591 (41.6)	589,164 (39.1)
	Married	23,266 (58.4)	919,725 (61.0)
Child's sex	Male	19,517 (51.0)	734,142 (51.4)
	Female	20,340 (49.0)	774,747 (48.7)
Residence	Copenhagen	9,366 (23.5)	387,774 (25.7)
	Big cities*	4,627 (11.6)	184,432 (12.2)
	Other	25,864 (64.9)	936,683 (62.1)

*Cities with over 100,000 residents.

We found that children exposed to bereavement during their prenatal life were more likely to be hospitalized with a type-1 diabetes diagnosis later in life (*see* *table 2*). This association was more pronounced when deaths were caused by a traumatic event and most significant for bereavement caused by death of a father or sibling (aIRR: 2.03, 1.22–3.38); the association was mainly seen for girls (aIRR: 2.91, 1.61–5.26). We did not detect significant effect measure modification for timing of exposure. We did attempt a matched sibling-pair analysis but the number of discordant type-1 diabetes pairs were too small to provide significant data for meaningful analysis.

**Table 2:** Incidence rate ratios and 95% CIs for Type-1 Diabetes (1979–2004).

			Exp./Unexp.d	IRR	aIRR*	Lower CI CL	Upper CI
Exp. to death of:		Father	4/5868	2.10	2.09	0.79	5.59
		Other child	31/5868	1.16	1.11	0.78	1.59
		Maternal gparent	116/5868	1.03	1.00	0.83	1.20
		Maternal uncle/aunt	2/5868	-	-	-	-
		Father/child	35/5868	1.23	1.18	1.18	1.64
		Father/child/gparent	151/5868	1.04	1.03	0.87	1.21
Traumatic death of:		Father	3/5868	2.46	2.51	0.81	7.80
		Other child	12/5868	1.94	1.94	1.10	3.42
		Maternal gparent	20/5868	1.04	0.98	0.63	1.51
		Maternal uncle/aunt	0/5868	-	-	-	-
		Father/child	15/5868	2.02	2.03	1.22	3.38
		Father/child/gparent	35/5868	1.31	1.25	0.89	1.74
Timing:	Preconception	Traumatic death	19/5868	1.08	1.04	0.66	1.63
		Non-traumatic	71/5868	0.94	0.93	0.74	1.18
	1^st^ Tri. (0–12 wks)	Traumatic death	4/5868	1.13	1.06	0.40	2.82
		Non-traumatic	15/5868	1.05	1.07	0.65	1.78
	2^nd^ Tri. (13–29 wks)	Traumatic death	6/5868	1.57	1.46	0.66	3.25
		Non-traumatic	18/5868	1.26	1.24	0.78	1.97
	3^rd^ Tri. (30–42 wks)	Traumatic death	6/5868	1.61	1.57	0.70	3.49
		Non-traumatic	14/5868	0.95	0.93	0.55	1.58
Sex (any kind of bereavement):	Male	Traumatic death	14/2981	0.98	0.90	0.52	1.53
		Non-traumatic	54/2981	0.90	0.89	0.68	1.17
	Female	Traumatic death	21/2887	1.47	1.46	0.95	2.24
		Non-traumatic	63/2887	1.07	1.07	0.83	1.37
Sex (father/sib bereavement only):	Male	Traumatic death	4/2981	1.11	1.10	0.41	2.94
		Non-traumatic	7/2981	0.67	0.66	0.31	1.38
	Female	Traumatic death	11/2887	2.87	2.91	1.61	5.26
		Non-traumatic	13/2887	1.22	1.10	0.64	1.89

*Adjusted for maternal age, residence, income, education, marital status, sibling order, calendar year, sex, parents' history of diabetes, gestational age at birth, birth weight.

## Discussion

To the best of our knowledge, this is the first population based study that has examined the link between prenatal stress exposure and diabetes in the offspring of humans. We found evidence to suggest that female fetuses exposed to maternal bereavement during the preconception and the prenatal period are at an increased risk for developing type-1 diabetes. We observed higher IRRs for traumatic deaths, especially those of a father or sibling. Since we do not have data on all stress exposures or biomarkers of stress hormones we cannot quantify the exposure contrast between the exposed and unexposed. However, we believe that the exposure contrast between the two groups is substantial as bereavement due to death of close family member is one of life's most stressful events. The difference seen for girls and boys could be due to chance or could be related to modification by sex hormones during prenatal life.

The main strengths of our study are the large cohort size, long follow-up time and high quality of data. Completeness of type-1 diabetes has been demonstrated in previous studies [13]. This study was made possible due to the vast information available in the Danish National Registers. Information in the CRS has been made available for research purposes by Danish legislation [14]. Outpatient data has been collected since 1995, though type-1 diabetes cases are most likely to be found via the in-patient hospital register. This is due to the disease having a sudden and aggressive onset and needing a thorough medical investigation. Today parental links for individuals born in Denmark since 1969 are considered accurate; Danish legislation requires all legal address changes be submitted to the CRS shortly after the move [15]. The main limitation of our study is the lack of inclusion of other sources of stressors and baseline measures of biological stress responses and measures of allostatic load. To get these data longitudinal biological sampling would be needed but it will at present be impossible for ethical and economic reasons. Another limitation in our study is the possibility of uncontrolled confounding. While we were able to control for sociodemographic factors such as education and income, as well as parents' history of diabetes, it is possible that other factors may confound the relationship between prenatal bereavement and development of type-1 diabetes. Lifestyle and background factors such as socioeconomic status may be partially controlled, but factors possibly related to type-1 diabetes such as smoking, maternal or child BMI may not. However, it is unclear clear why these factors should be related to bereavement, and our data indicate that in this population death of a close family member affect all segments of the population equally.

The etiology of type-1 diabetes and the timing of a causal window is not clearly understood, though the disease is postulated to have a multiple stages. It is possible that bereavement is one of the environmental factors that when combined with a genetic factor initiates the disease process shortly after conception. Type-1 diabetes is a rare disease in childhood and if stress exposure can cause the disease one would not expect common stressors to play a role. The disease could on the other hand be caused by a common cause if that cause produces susceptibility for a second hit later in life; should this be the case one would expect low to moderate association for the prenatal exposure if data were not stratified on the second set of causes. The association between prenatal bereavement and type-1 diabetes was observed mainly among female children, but not among male children, exposed to traumatic father or sibling deaths. Previous studies have demonstrated important sex differences in glucocorticoid programming of the HPA axis and metabolic physiology, but how these differences contribute to differential risk for type-1 diabetes is unknown [16], [17].

This study supports the view that type-1 diabetes may have causes that operate in fetal life. We have focused on stress as one of many possible exposures but are aware that stress may just be an upstream cause of other factors of importance such as infections, or changes in dietary habits. Stress exposure may impact the immune system and thus increase the susceptibility of infections. If stress is not a direct cause of diabetes it would explain the rather weak association we find.

While one cannot avoid bereavement as a potential stressor during pregnancy one may be able to avoid other sources of stress, or mitigate the effects via therapy. This study and other studies suggest that exposure to severe stress during the critical period that surrounds pregnancy may have life-long effects on the offspring. We did not find a strong link between bereavement and diabetes when the death was non-traumatic or that of a grandparent or maternal sibling, suggesting that only the most severe stress exposures have a programming effect. A small sample human study with a positive finding has been published on markers of glucose metabolism also suggested a link between stress and diabetes [18].

The findings of this study may have public health implications especially in settings where sources of intense stress are increasing, such as in areas of political unrest, migration, conflict or environmental disasters. Future research should consist include a large enough sample size to assess affects of timing of exposure and also corroborate the findings in this study.
